# Comparison of efficacy and safety of adjuvant therapies versus sorafenib in hepatocellular carcinoma: a systematic review and network meta-analysis

**DOI:** 10.3389/fphar.2025.1502931

**Published:** 2025-03-03

**Authors:** Wenjun Quan, Hanifah Fazlin Zulkifli, Norhafizah Saari, Rafidah Hanim Shueb, Nazri Mustaffa

**Affiliations:** ^1^ Department of Medicine, School of Medical Sciences, Universiti Sains Malaysia, Kelantan, Malaysia; ^2^ Department of chemical pathology, School of Medical Sciences, USM, Kelantan, Malaysia; ^3^ IC-Innovation in Advanced Material and Photonics, Advanced Materials Research Centre (AMREC), SIRIM Berhad, Kulim, Kedah, Malaysia; ^4^ Department of Medical Microbiology and Parasitology, School of Medical Sciences, University Sains Malaysia, Kelantan, Malaysia; ^5^ Institute for Research in Molecular Medicine (INFORMM), Universiti Sains Malaysia, Kelantan, Malaysia; ^6^ Department of Medicine, Hospital University Sains Malaysia, Kelantan, Malaysia

**Keywords:** hepatocellular carcinoma, sorafenib, adjuvant therapy, meta-analysis, systematic review

## Abstract

**Purpose:**

Diverse novel therapeutic options for hepatocellular carcinoma (HCC) have surfaced in recent years. However, it is increasingly difficult to select the optimal medication. This research aims to assess overall survival (OS), progression-free survival (PFS), objective response rate (ORR), disease control rate (DCR), adverse events (AEs), and severe adverse events (SAEs) in HCC patients receiving adjuvant therapies compared to those receiving sorafenib.

**Methods:**

Four databases were used to search articles. Only randomized controlled trials were included. Indicators such as OS, PFS, DCR, ORR, AEs and SAEs were used as outcomes. The protocol for this meta-analysis was registered with PROSPERO (Registration ID: CRD42024544394).

**Results:**

Forty trials were included in this meta-analysis. The Oxaliplatin, Fluorouracil, and Leucovorin (OFL) + sorafenib group and the sintilimab + bevacizumab biosimilar group decreased the risk of death and increased PFS, ORR, and DCR. Yet, they also yielded remarkable adverse effects and severe adverse effects. To sum up, the atezolizumab + bevacizumab combination and tepotinib were recommended due to their favorable performance on all indexes.

**Conclusion:**

This study further substantiates the efficacy of combination therapies in HCC, while they cause more toxicity in general. It is pressingly urgent to develop new drugs for liver cancer and find rational strategies to alleviate AEs.

**Systematic Review Registration:**

PROSPERO, identifier CRD42024544394.

## 1 Introduction

HCC is the most predominant form of liver cancer and the third leading contributor to global cancer-related deaths, resulting in over 700,000 deaths each year (Kim et al., 2020). The global incidence of HCC is increasing, particularly in East Asia and sub-Saharan Africa, where chronic hepatitis B and C infections are prevalent, affecting over 250 million and 71 million people, respectively. (Sagnelli et al., 2020; Yau et al., 2019) Despite advances in screening and diagnostic techniques, many patients are diagnosed at advanced stages, significantly limiting the availability of therapeutic options. This challenge underscores the pressing importance of achieving the United Nations Sustainable Development Goal 3 (SDG 3), which strives to ensure healthy lives and wellbeing for all individuals. Specifically, SDG 3.4 aims to reduce premature mortality from non-communicable diseases (including cancer) by one-third, by 2030 through preventive measures, treatment, and the promotion of mental health and wellbeing. (Targets of Sustainable Development Goal 3 (who.int))

The current therapeutic options for HCC encompass surgical resection, liver transplantation, and loco-regional therapies [e.g., conventional transarterial chemoembolization (cTACE), and radiofrequency ablation] (Coffman-D’Annibale et al., 2023). Nonetheless, systemic therapies are indispensable for patients with advanced or unresectable HCC. Sorafenib, an oral multikinase inhibitor, has been established as the first choice for advanced HCC since its approval in 2007. It notably enhances OS compared to placebo, with median OS increasing from 7.9 months to 10.7 months in landmark clinical trials (Marisi et al., 2018). Nevertheless, the efficacy of sorafenib is modest, accompanied by considerable AEs like diarrhea, hand-foot skin reaction, and hypertension, highlighting the search for more effective and safer therapeutic alternatives (Ai et al., 2019). Recent advances have paved the way for new systemic therapies, including lenvatinib (with comparable efficacy to sorafenib) and immune checkpoint inhibitors (ICIs) (e.g., nivolumab and pembrolizumab), providing encouraging outcomes in response rates and OS (Ntellas and Chau, 2024). Given the evolving therapeutic landscape, ongoing research is essential to refine treatment approaches and clinical outcomes for patients with advanced HCC.

Adjuvant therapy, a supplementary treatment given after primary therapy, is to diminish the likelihood of cancer recurrence and enhance outcomes in HCC. Recent clinical trials have highlighted the potential effectiveness of various adjuvant strategies, including targeted therapies, ICIs, and combination regimens. For instance, ICIs such as nivolumab and pembrolizumab have demonstrated favorable response rates and OS (Onoi et al., 2020). Similarly, targeted therapies like sorafenib and lenvatinib have the potential to delay disease recurrence. Despite these advances, the optimal adjuvant treatment remains unclear. Trials are currently underway to explore the efficacy and safety of different therapeutic combinations (Yang et al., 2024). Llovet et al. (2024) Therefore, further research is required to establish evidence-based guidelines for adjuvant therapy in HCC.

Several meta-analyses have synthesized available evidence on the efficacy and safety of different therapeutic options for HCC, (Ntellas and Chau, 2024; Wu et al., 2023; Fulgenzi et al., 2023; Tian et al., 2018; Wang et al., 2020; Li et al., 2023) but they are mostly limited to pairwise comparisons, restricting comprehensive assessment. Network meta-analysis (NMA) overcomes this limitation by integrating direct and indirect evidence from multiple randomized controlled trials (RCTs), allowing for concurrent and comprehensive assessment of numerous interventions against a common comparator. This systematic review and NMA aims to fill the gap by comparing the efficacy and safety of other adjuvant therapies versus sorafenib in HCC, provide an evidence-based understanding of treatment effectiveness, and point out future research directions for HCC management. Additionally, the systematic review will synthesize current research on HCC, focusing on epidemiology, risk factors, and advances in diagnostic and therapeutic approaches. In the context of SDG 3, the study aims to enhance the overall comprehension of HCC management worldwide.

## 2 Materials and methods

### 2.1 Meta-analysis registration

This NMA followed the PRISMA guidelines, and the protocol was registered with PROSPERO (Registration ID: CRD42024544394).

### 2.2 Literature search

PubMed, EMBASE, Web of Science, and Cochrane Library were searched for related clinical trials until January 2024. The search strategies are displayed in Supplementary Table S1.

### 2.3 Selection criteria

Inclusion criteria:

1) Patients diagnosed with HCC
2) RCTs
3) Participants treated with any adjuvant therapy versus sorafenib
4) Reporting at least one of the following outcomes: OS, PFS, ORR, DCR, AEs, and SAEs.

Exclusion criteria:

1) Non-original articles
2) Studies without the available full text
3) Case reports, conference abstracts, reviews, short surveys, or expert opinions
4) Animal trials
5) Studies without a control group
6) Participants treated with placebo or best supportive care versus sorafenib.

### 2.4 Data extraction

Two authors independently identified the eligible papers based on inclusion and exclusion criteria and subsequently extracted data. Any discrepancies or disagreements during the process were solved via discussion with a third author. The data extracted from every article encompassed basic study characteristics (author, year, country, sample size, age, intervention, and outcome measures); hazard ratios (HRs) and 95% confidence intervals (CIs) for outcome measures (OS, PFS); available outcomes in terms of ORR, DCR, AEs, and SAEs. Furthermore, HRs and 95% CIs were calculated from the Kaplan-Meier curves using WebPlotDigitizer (https://apps.automeris.io/wpd/index.zh_CN.html) and HR data converter.

### 2.5 Quality assessment

Cochrane risk of bias assessment tool was employed by two reviewers to judge the quality of enrolled RCTs independently, with a third reviewer deciding the conflicting items. Seven items, including random sequence generation, allocation concealment, blinding of participants and personnel, blinding of outcome assessment, incomplete outcome data, selective reporting, and other biases were assessed and graded as high, low, or unclear risk of bias. All scores were entered into Review Manager 5.4 to generate images. The assessment results are displayed in Supplementary Figure S1.

### 2.6 Statistical methods

HRs with corresponding 95% CIs were log-transformed and entered into R version 4.3.2 to estimate OS and PFS. R version 4.3.2 was also used to assess ORR, DCR, AEs, and SAEs. Then, league tables, forest plots, surface under the cumulative ranking (SUCRA) values, and SUCEA curves were generated to compare various adjuvant therapies. In forest plots, the numerical range of different indicators reflected the effectiveness and safety of the treatment. For OS and PFS, if the value range was <1, it indicated that the experimental group had a longer survival time, suggesting that the treatment may be effective; if the value range was >1, it suggested that the survival time in the experimental group was shorter, indicating the treatment may be ineffective or even harmful. For ORR and DCR, if the value range was >1, it meant the ORR or DRR was higher in the experimental group, indicating better treatment efficacy; if the value range was <1, the control group showed better outcomes. For AEs and SAEs, if the value range was >1, it suggested a higher incidence of AEs in the experimental group, indicating greater toxicity; if the value range was <1, the experimental group yielded fewer AEs, suggesting better safety. Furthermore, SUCRA values were imported to Prism version 9 to generate bar charts. *I* ^*2*^ statistic was utilized to quantify heterogeneity (Higgins et al., 2003). The random-effects and fixed-effects models were adopted based on *I* ^2^ values. *I* ^2^ values <50% implied low heterogeneity and >50% implied considerable heterogeneity among the studies. Furthermore, the data for all outcome measures were imported into Stata SE version 15 to create network plots and meta-funnel plots for assessing publication bias.

## 3 Results

### 3.1 Study selection

**FIGURE 1:**
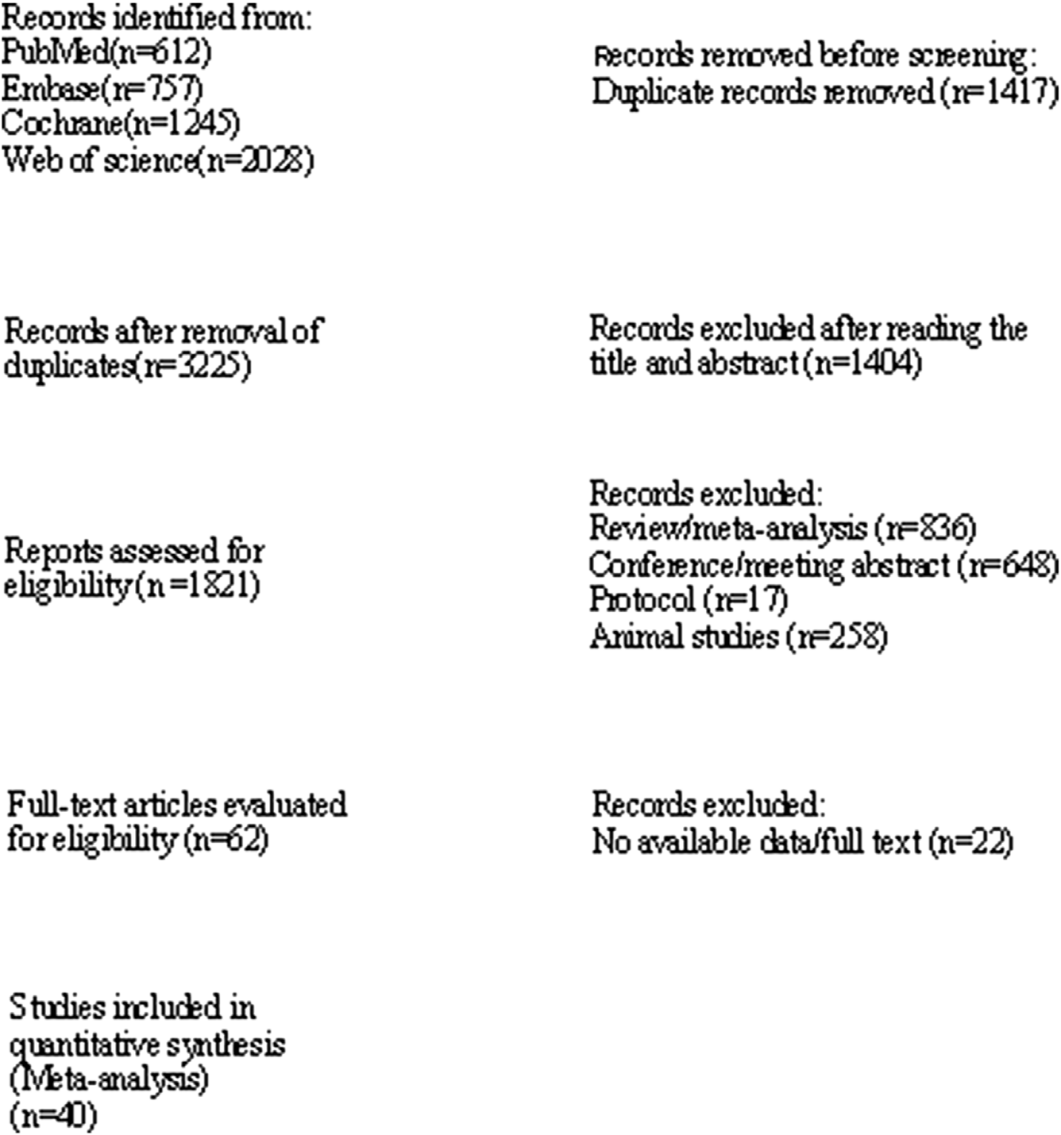
Flowchart of the study selection process.

4,642 records were identified, of which 1,417 duplicate records were removed and 1,404 were excluded after reading the titles and abstracts. Then, 1759 records were further excluded as they were reviews, meta-analyses, conferences, meeting abstracts, protocols, and animal studies. Finally, 40 records were enrolled after 22 records were removed due to the lack of available data or full texts (Abdel-Rahman et al., 2013; Abou-Alfa et al., 2019; Assenat et al., 2019; Blanc et al., 2021; Cainap et al., 2015; Cheng et al., 2015; Cheng et al., 2013; Cheng et al., 2016; Chow et al., 2018; Ciuleanu et al., 2016; El Shorbagy et al., 2021; Finn et al., 2020; Giorgio et al., 2016; Haruna et al., 2021; He et al., 2019; Ikeda et al., 2016; Johnson et al., 2013; Jouve et al., 2019; Kelley et al., 2022; Koeberle et al., 2016; Kondo et al., 2019; Kudo et al., 2018; Lee et al., 2016; Liang and Hu, 2020; Park et al., 2019; Ren et al., 2021). The study selection process is exhibited in Figure 1.

### 3.2 Baseline characteristics

These articles (with 12,415 participants) were published between 2012 and 2022 and conducted in the United States, France, Egypt, Romania, China, Singapore, Italy, Japan, Kashiwa, UK, Switzerland, Korea, and Germany. The dosage of sorafenib (400 mg twice daily) was consistent in most articles. The details of the included RCTs are displayed in Supplementary Table S2. The thickness of the lines represents the number of studies or the sample size of comparisons in the network plots. Thicker lines indicate more studies or larger sample sizes. For example, the OS, ORR, and DCR network plots showed that multiple studies directly compared hepatic arterial infusion chemotherapy (HAIC) + sorafenib versus sorafenib single agent; the AEs network plots showed that more research compared Nivolumab versus Sorafenib (Supplementary Figure S2).

### 3.3 Primary outcomes

#### 3.3.1 OS

**FIGURE 2:**
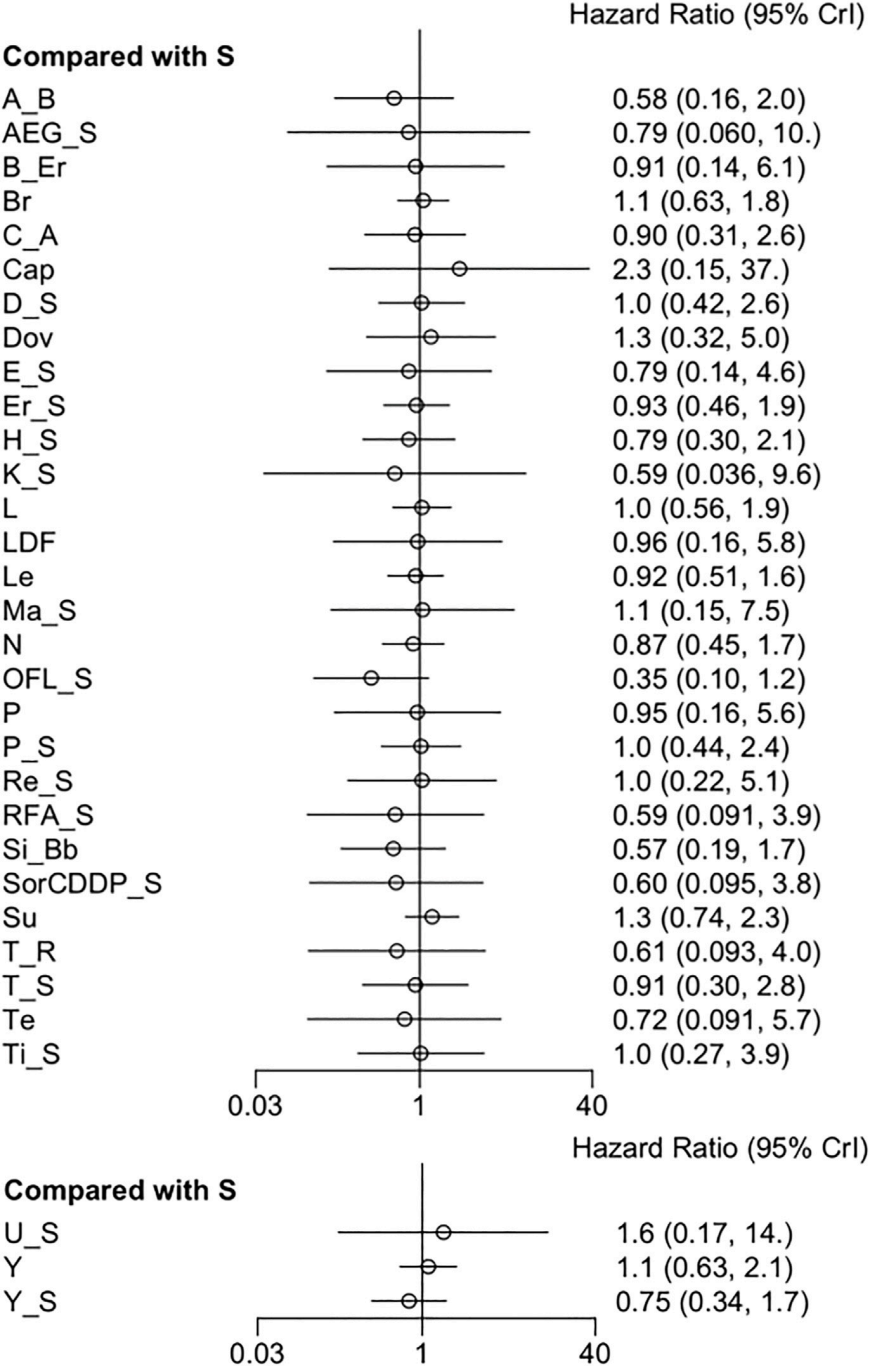
Forest plot on OS.

Thirty-eight RCTs provided data for OS. The pooled results revealed that all adjuvant therapies had no significant difference in OS (Figure 2) (Supplementary Table S3). No considerable heterogeneity was observed (*I* ^*2*^ = 0%). However, sorafenib + OFL (Oxaliplatin, Fluorouracil, and Leucovorin) outperformed other measures in prolonging OS, followed by sintilimab + a bevacizumab biosimilar and atezolizumab + bevacizumab (Supplementary Figure S3). The results regarding OS demonstrate that certain combination therapies, such as sorafenib + OFL, were superior in increasing OS to other treatment regimens. This finding may be attributed to several factors. First, the combination of sorafenib with OFL could produce a synergistic effect. Sorafenib, a targeted therapy, inhibits multiple molecular pathways involved in tumor progression and angiogenesis. OFL, a chemotherapy regimen, targets rapidly dividing cancer cells. This dual mechanism of action might enhance therapeutic efficacy and improve survival outcomes (He et al., 2019). Moreover, this finding aligns with earlier studies showing that combination therapies tend to offer better outcomes than single-agent treatments in various cancers, including HCC (Katsanos et al., 2017).

#### 3.3.2 PFS

**FIGURE 3:**
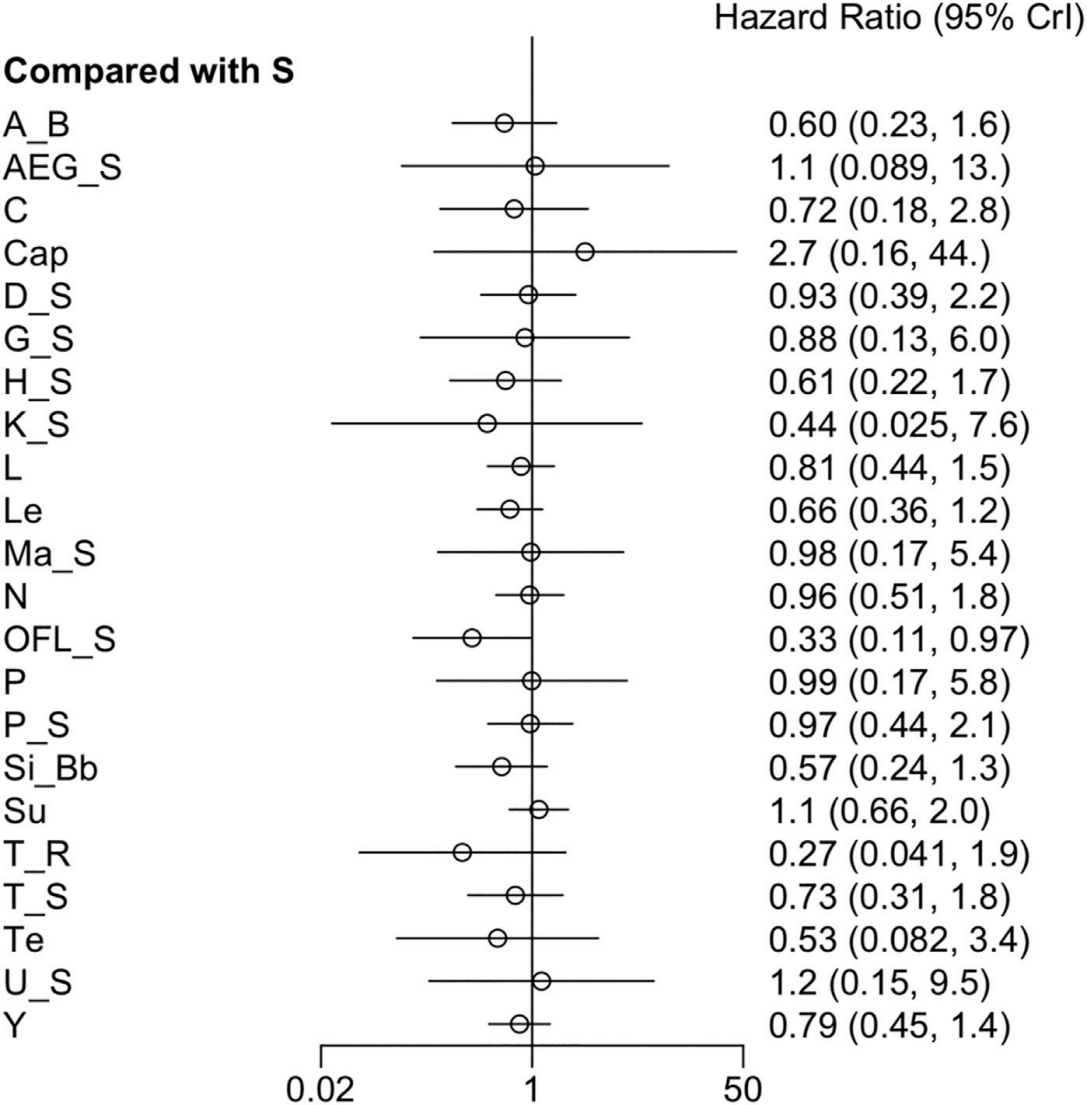
Forest plot on PFS.

Twenty-six RCTs reported a correlation between adjuvant therapies and PFS. The forest plot and league table indicated that sorafenib + OFL was superior in controlling HCC progression to sorafenib (Figure 3) (Supplementary Table S4). Moreover, the league table showed that sorafenib + OFL was superior to sunitinib. The data were assessed using a fixed-effects model, and *I* ^2^ value for heterogeneity was 0%. Furthermore, sorafenib + OFL, cTACE + radiotherapy, and sintilimab + a bevacizumab biosimilar were slightly more effective than other therapies in delaying disease progression (Supplementary Figure S4).

#### 3.3.3 ORR

**FIGURE 4:**
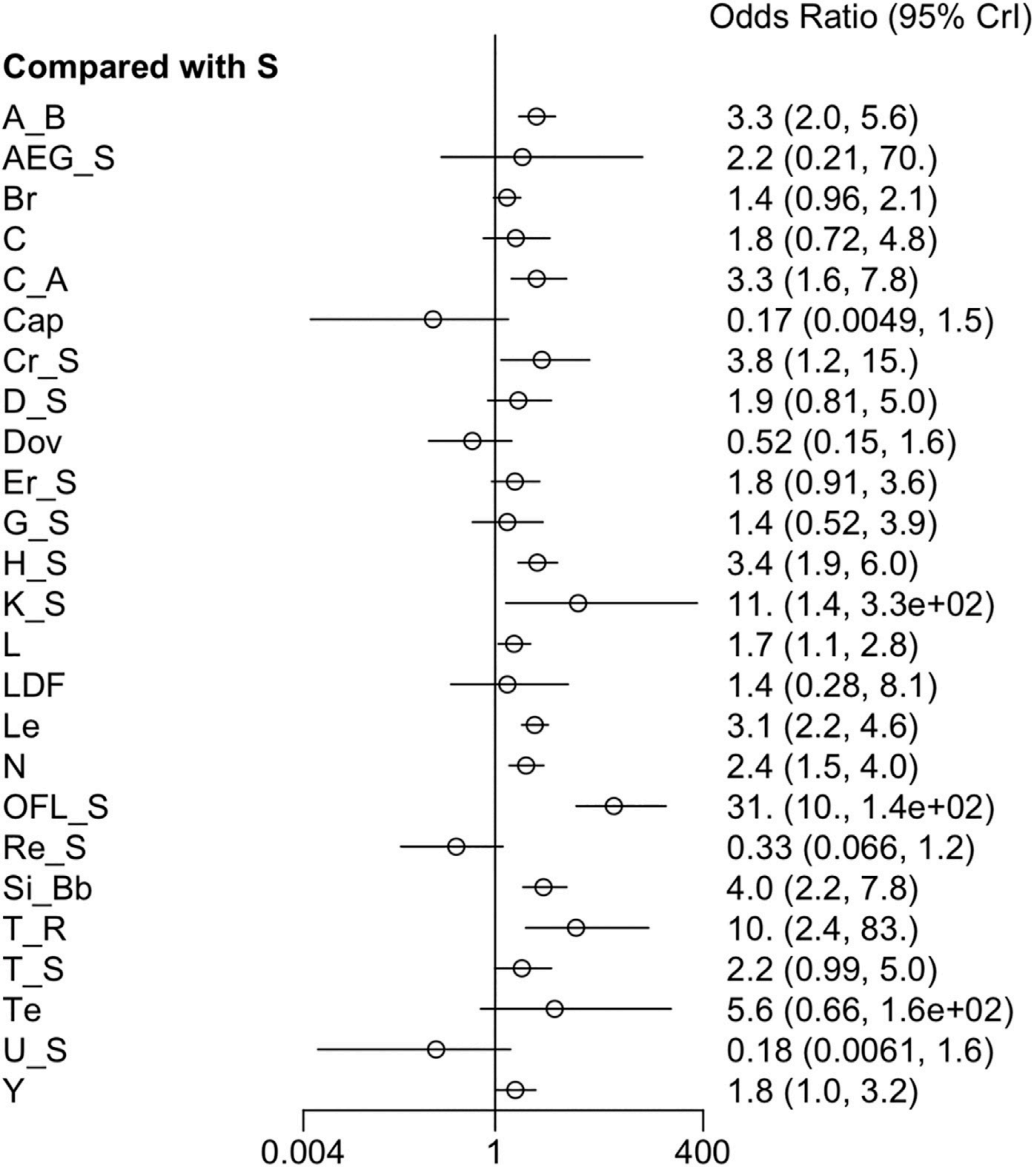
Forest plot on ORR.

Twenty-seven RCTs reported ORR. The forest plot and league table showed that, compared to sorafenib, atezolizumab + bevacizumab, cabozantinib + atezolizumab, cryotherapy + sorafenib, HAIC + sorafenib, vitamin K + sorafenib, linifanib, lenvatinib, nivolumab, sorafenib + OFL, sintilimab + a bevacizumab biosimilar, and cTACE + radiotherapy yielded higher ORR (Figure 4). Additionally, the league table manifested that sorafenib + OFL greatly improved ORR compared to most therapies, except for AEG35156 + sorafenib, vitamin K + sorafenib, cTACE + radiotherapy, and tepotinib (Supplementary Table S5). Given low heterogeneity (*I* ^*2*^ = 11%), a fixed-effects model was applied. Moreover, sorafenib + OFL yielded the highest ORR based on the SUCRA ranking (SUCRA, 97.5%), followed by cTACE + radiotherapy (SUCRA, 89.0%) (Supplementary Figure S5).

#### 3.3.4 DCR

**FIGURE 5:**
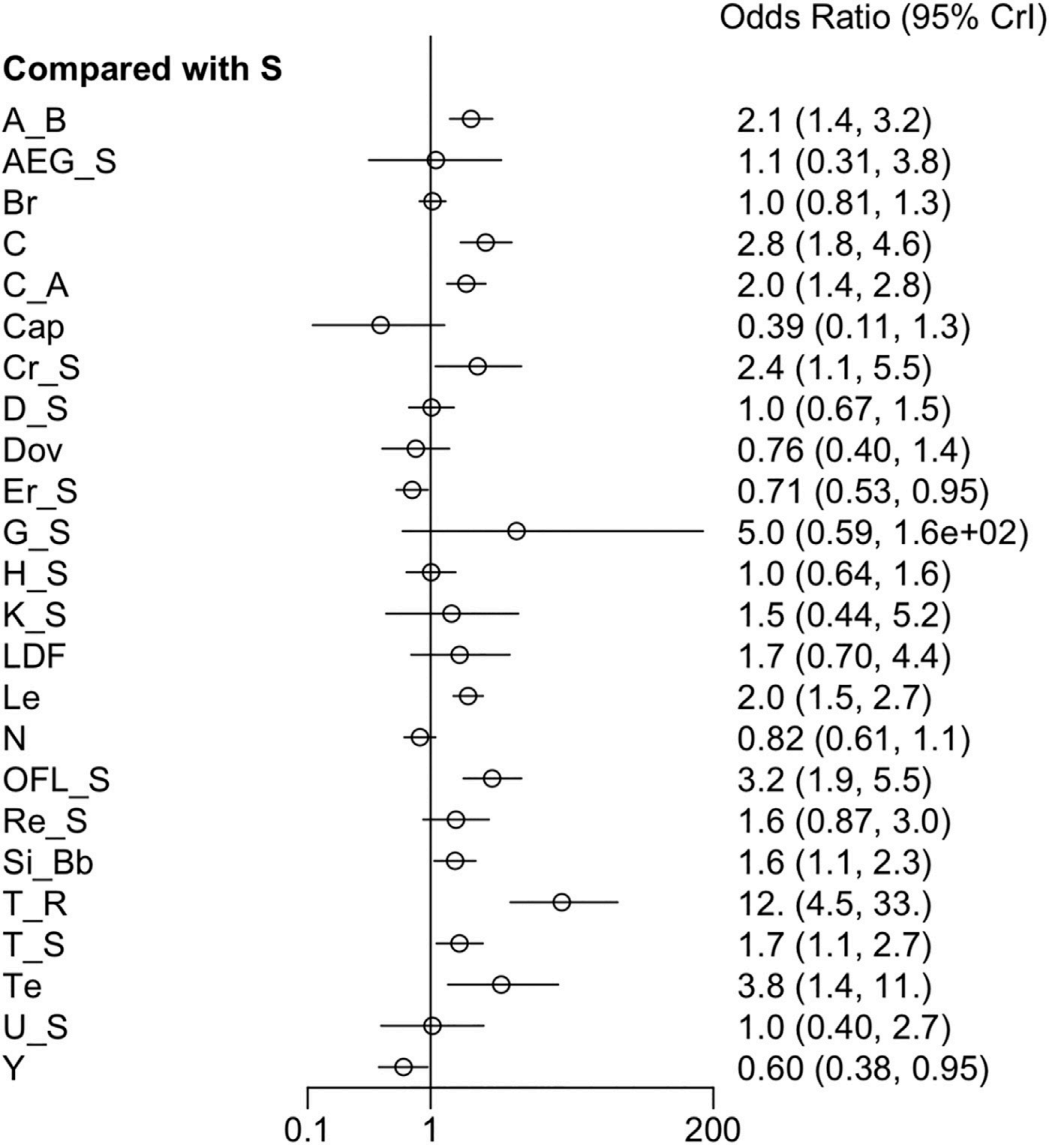
Forest plot on DCR.

Twenty-six RCTs reported DCR. The pooled analysis noted that DCR was remarkably higher in patients treated with atezolizumab + bevacizumab, cabozantinib, cabozantinib + atezolizumab, cryotherapy + sorafenib, lenvatinib, sorafenib + OFL, sintilimab + a bevacizumab biosimilar, cTACE + radiotherapy, cTACE + sorafenib, and tepotinib compared to those receiving sorafenib only. In contrast, DCR was notably lower in the erlotinib + sorafenib group than in the sorafenib group (Figure 5). Moreover, a fixed-effects model was used due to low heterogeneity (*I* ^*2*^ = 25%). The league table reported that cTACE + radiotherapy yielded the highest DCR over all adjuvant therapies except for GEMOX + sorafenib and tepotinib (Supplementary Table S6). Tepotinib ranked second only to cTACE + radiotherapy, with a slight advantage over OFL + sorafenib (Supplementary Figure S6).

#### 3.3.5 AEs

**FIGURE 6:**
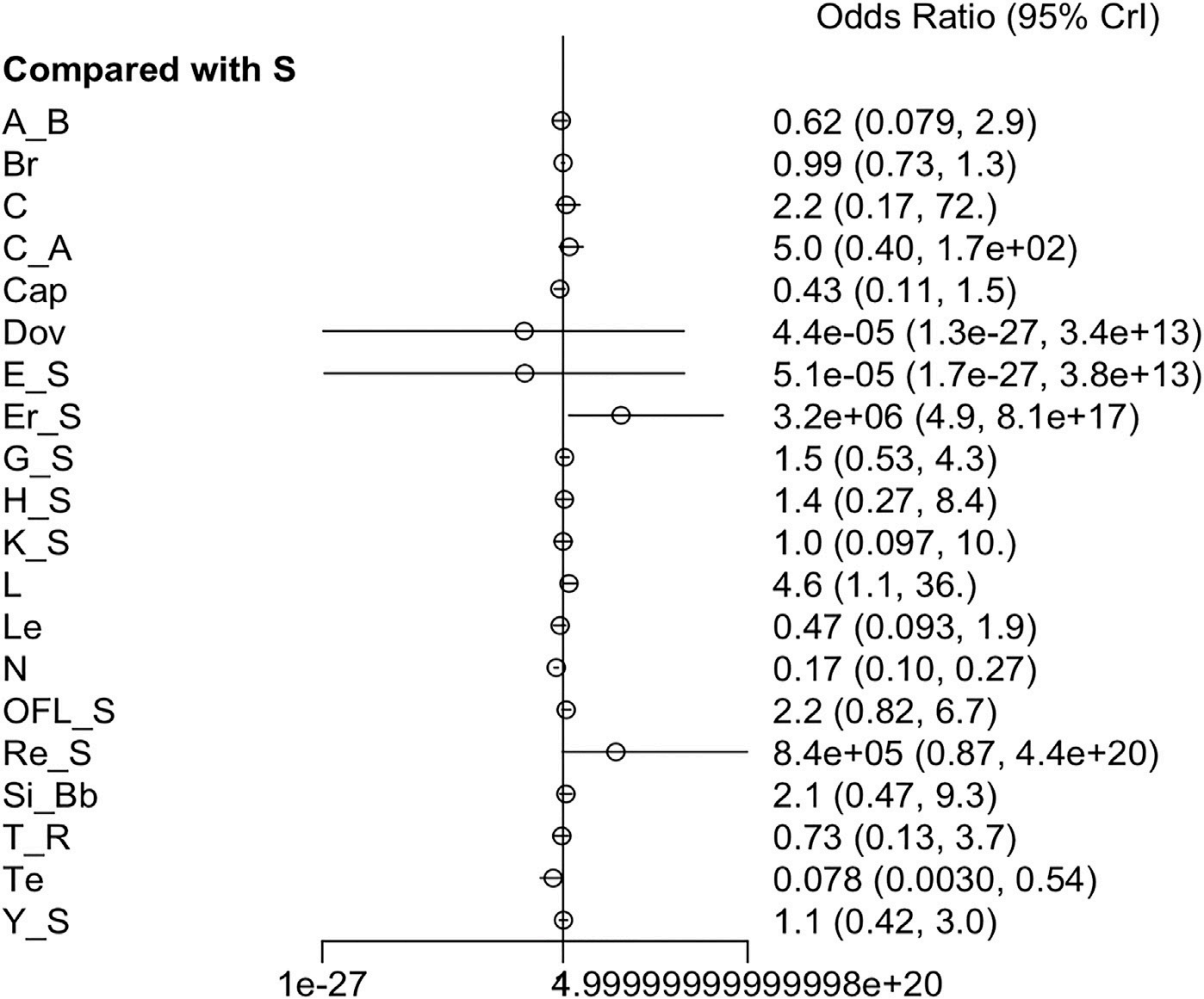
Forest plot on AEs.

Twenty-one RCTs offered available data on AEs, and twenty-three RCTs offered data on SAEs. Nivolumab appeared to have better safety profiles than sorafenib single agent. Erlotinib + sorafenib and linifanib resulted in a higher risk of AEs than sorafenib single agent. Moreover, the incidence of grade ≥3 AEs was prominently higher in cabozantinib, cabozantinib + atezolizumab, everolimus + sorafenib, HAIC + sorafenib, linifanib, lenvatinib, and 90Y loaded resin microspheres + sorafenib groups than sorafenib single agent (Figure 6) (Supplementary Figure S7). Low heterogeneity was detected (*I* ^*2*^ = 0%) in the analysis, and a fixed-effects model was adopted. The league table indicated that sorafenib + OFL results in more AEs than cTACE + radiotherapy, GEMOX + sorafenib, nivolumab, and tepotinib. Additionally, sorafenib + OFL caused more SAEs than capecitabine, nivolumab, and tepotinib (Supplementary Tables S7, S8). Regarding treatment safety, tepotinib ranked first (SUCRA, 90.4%), followed by nivolumab (SUCRA, 87.4%) and capecitabine (SUCRA, 73.1%) (Supplementary Figure S8). However, GEMOX + sorafenib (SUCRA, 97.5%), nivolumab (SUCRA, 93.3%), and tepotinib (SUCRA, 84.8%) showed the lowest incidence of SAEs (Supplementary Figure S9).

AEs can significantly impact the quality of life of patients, thus worsening symptoms, limiting daily activities, and even reducing treatment adherence. Additionally, certain treatment regimens, such as sorafenib + OFL, showed a higher incidence of AEs, which may affect their clinical acceptability. Therefore, exploring strategies to mitigate toxicity is crucial. Optimizing dose adjustments, incorporating supportive therapies (such as hepatoprotective agents and hematopoietic growth factors), selecting patients who are suitable for intensive treatment, and monitoring and managing specific types of toxicity (such as liver dysfunction or hematologic toxicity) may be beneficial to reducing toxicity.

### 3.4 Publication bias

The funnel plots for OS, PFS, ORR, DCR, AEs, and SAEs (Supplementary Figure S10–S15) were symmetrical, suggesting no or limited publication bias.

## 4 Discussion

HCC is a prominent contributor to cancer-related mortality globally, with poor prognoses in advanced cases (Chuma et al., 2015). Despite curative options for early-stage HCC, most patients are diagnosed at advanced stages and cannot benefit from surgical interventions. Chronic liver disease, a major risk factor for HCC, further complicates and limits treatment options. Novel targeted and immunotherapeutic approaches have been developed as a result of recent advances in understanding molecular drivers of HCC pathogenesis and the complex interplay between the tumor and its microenvironment (Zhao et al., 2023). Hence, a comprehensive assessment of current adjuvant therapies is necessary. This NMA compared all adjuvant therapies with sorafenib and offered valuable guidance for clinicians on medication administration.

Sorafenib, a multi-kinase inhibitor targeting VEGFR, PDGFR, and RAF, is the first systemic therapy to improve OS in advanced HCC patients (Chuma et al., 2015). However, the frequent development of resistance to sorafenib emphasizes the necessity for alternative therapeutic options. Several other tyrosine kinase inhibitors, including regorafenib, lenvatinib, and cabozantinib, have been approved for advanced HCC since they can prolong OS. (Chuma et al., 2015; Gong et al., 2020) These agents target diverse signaling pathways involved in HCC pathogenesis, including angiogenesis, cell proliferation, and survival. ICIs like nivolumab and pembrolizumab significantly enrich the therapeutic choices for advanced HCC (Fessas et al., 2020).

The efficacy of adjuvant therapies versus sorafenib was evaluated based on scores in OS, PFS, ORR, DCR, AEs, and SAEs. OS, the primary endpoint, indicates the overall efficacy of treatments in prolonging patients’ survival. Additionally, high PFS and ORR scores signify preferable outcomes by delaying disease progression and reducing tumor size post-treatment. These scores can offer initial clues to treatment efficacy. We selected RCTs on patients treated with adjuvant therapies and included any reported data on OS, PFS, ORR, DCR, AEs, and SAEs.

NMA results noted that combined adjuvant therapies significantly prolonged OS, PFS, ORR, and DCR, suggesting their efficacy in delaying HCC progression. Moreover, multiple inhibitors may reduce the risk of resistance compared to single agents. Meanwhile, safety measures are needed to determine the overall efficacy. The safety of adjuvant therapies was evaluated based on AEs and SAEs. Notable AEs and SAEs were revealed in patients treated with various combined therapies, including cabozantinib + atezolizumab, HAIC + sorafenib, erlotinib + sorafenib, and sorafenib + OFL.

Due to potential SAEs such as gastrointestinal effects, myelosuppression, hepatoxicity, liver dysfunction, cardiovascular effects, and thrombocytopenia, these combination therapies are not recommended (Tabernero et al., 2013). Consequently, their clinical application is limited due to increased toxicity concerns. Tepotinib demonstrated the highest SUCRA score (90.4%) of AEs, followed by nivolumab (SUCRA 87.4%) and capecitabine (SUCRA 73.1%). Despite their high safety ranking, they may carry potential AEs. It is notable to strike a balance between safety and efficacy to minimize treatment interruptions. Therefore, it is necessary to consider drug toxicity and minimize AEs while using combination therapy.

Based on a comprehensive scoring evaluation, cTACE + radiotherapy performed well on every index. For early-stage HCC patients, cTACE + radiotherapy was effective, especially for unresectable tumors. However, patients with intermediate to advanced HCC typically received systemic therapy as the standard first-line treatment. In some cases, cTACE + radiotherapy can be concurrently used with systemic therapy. The combination of atezolizumab and bevacizumab demonstrated potent efficacy as a first-line therapy for HCC, surpassing sorafenib monotherapy in safety metrics (OS: 67.8%, PFS: 62.5%, ORR: 69.2%, DCR: 70.1%, AEs: 64.9%, SAEs: 64.9%). In the ongoing IMbrave05 clinical trial for advanced HCC patients, this regimen substantially improved recurrence-free survival (RFS) (Qin et al., 2023). Despite the promising advantage in enhancing RFS, additional detailed analyses are warranted to optimize dosing and ensure safety. Tepotinib ranked second in overall scores (OS: 56.4%, PFS: 62.7%, ORR: 73.9%, DCR: 85.6%, AEs: 90.4%, SAEs: 84.8%) and was considered safe for clinical application. Although sorafenib + OFL is associated with AEs, its high efficacy scores (OS: 83.4%, PFS: 83.2%, ORR: 97.5%, DCR: 84.8%, AEs: 39.2%, SAEs: 37.2%) suggest potential application for clinical practice. Therefore, clinicians should pay special attention to AEs when using the OFL + sorafenib combination therapy. Additionally, the phase II trial SECOX (sorafenib, capecitabine, and oxaliplatin) reported promising outcomes (OS: 11.73 months, PFS: 5.29 months) with minimal AEs, underscoring its efficacy in HCC treatment (Yau et al., 2013). Nivolumab also demonstrated favorable overall scores (OS: 52.5%, PFS: 38.4%, ORR: 55.9%, DCR: 19.6%, AEs: 87.4%, SAEs: 93.3%) and received FDA approval for HCC therapy, highlighting the feasibility and efficacy of ICIs.

The safety and toxicity profiles of various therapies differ significantly, making it essential for clinicians to strike a balance between efficacy and potential AEs. Identifying patient subgroups that may benefit from combination therapies is particularly valuable. Patients with advanced HCC, preserved performance, or specific molecular characteristics (e.g., high angiogenic activity) may derive more therapeutic benefits from certain combination therapies. Moreover, clinicians must carefully assess efficacy versus toxicity by considering key factors, including liver function (Child-Pugh score), comorbidities, and prior treatment history. Implementing strategies such as dose optimization, toxicity monitoring, and timely management for AEs can significantly enhance efficacy while maintaining an acceptable safety profile. The studies reported low heterogeneity (I^2^ < 50%) for most outcomes. Differences in populations (e.g., disease stage, liver function, prior treatments), study design (e.g., follow-up duration, outcome assessment methods), and treatment regimen (e.g., dosage, combination therapies) may contribute to residual heterogeneity. Specifically, the RCTs included in this study shared similar methodologies, randomization strategies, and data analysis approaches, thereby minimizing differences across studies. Standardized measurement of OS, PFS, and AEs enhances result comparability and reduces variability due to measurement errors. These compared treatment regimens, including sorafenib and other adjuvant therapies, exhibit consistency in dosage, treatment protocols, and follow-up durations, which further lower treatment-related heterogeneity. Moreover, the screening process may have excluded studies with significant methodological differences, ensuring greater methodological consistency among the included studies.

Funnel plot is a graphical tool used to show the relationship between sample size and effect size for studies included in a meta-analysis, which helps investigate potential publication bias (Biljana et al., 1999). In our study, the funnel plots suggested no significant publication bias. However, funnel plots have inherent limitations in detecting publication bias. Firstly, their sensitivity is reduced when the number of studies is small, which makes accurate identification of bias more challenging. Secondly, asymmetry in the funnel plot may not fully indicate publication bias; it may also be attributed to small-study effects, where smaller-sample studies tend to report more exaggerated treatment effects. Furthermore, heterogeneity across studies, such as differences in study design, patient characteristics, and treatment interventions, can influence the symmetry of the funnel plot, thus complicating the distinction between true bias and natural variability. Therefore, to comprehensively assess publication bias, funnel plots should be considered alongside other statistical methods, such as Egger’s test or comparison-adjusted funnel plots (Lin, 2019).

Compared with previous meta-analyses, this NMA provides a more comprehensive analysis of current adjuvant therapies. It is crucial to consider concurrent conditions such as liver cirrhosis, impaired liver function, hepatitis B virus infection, and diabetes when choosing optimal combination therapies for HCC patients. Further extensive research with larger sample sizes is required to thoroughly assess overall treatment safety and tolerability profiles (Du et al., 2024). The lack of basic information on patients, including liver cancer stage and hepatitis B infection prevents us from conducting subgroup analysis. Moreover, the lack of RCTs and the variability among trials may introduce biases and influence the interpretation of the findings.

## 5 Conclusion

The NMA illustrates that the combination therapy of OFL + sorafenib has advantages in OS, PFS, and ORR over other adjuvant therapies; cTACE + radiotherapy has a superior DCR than other adjuvant therapies; the safety profile of tepotinib is better than other adjuvant therapies. However, based on efficacy and safety, the atezolizumab + bevacizumab combination should be the most appropriate and promising adjuvant therapy. Future clinical practice guidelines can consider the atezolizumab + bevacizumab combination as one of the standard treatment options. The long-term efficacy and safety of this combination therapy should be validated in further research.

Given the limitations, further large-sample and high-quality RTCs are necessary for validation in the future. Additionally, future RCTs should focus on comprehensive patient data, including disease staging, classification, HBV/HCV infection status, and liver function (Child-Pugh score) to assess the efficacy of different treatment regimens in these subgroups. Given the high prevalence of comorbidities such as diabetes and hypertension, future studies should specifically include these populations to evaluate both the efficacy and safety of treatments. Lastly, given the promising potential of nanomedicine and herbal combination therapies, future research should investigate whether these innovative approaches can enhance therapeutic efficacy while minimizing toxicity.

## Data Availability

The original contributions presented in the study are included in the article/Supplementary Material, further inquiries can be directed to the corresponding author.

## Glossary

S: Sorafenib
Cap: Capecitabine
D: Doxorubicin
G: GEMOX (gemcitabine+oxalipatin)
U: UFT (tegafur-uracil)
P: Pravastain
L: Linifanib
Su: Sunitinib
Dov: Dovitinib
Ti: Tigatuzumab
Y: 90Y loaded resin microspheres
Ma: Mapatumumab
M: Metformin
A: Atezolizumab
B: Bevacizumab
K: vitamin K
OFL: Oxaliplatin, Fluorouracil, and Leucovorin
SorCDDP: hepatic arterial infusion chemotherapy with cisplatin
Br: Brivanib
C: Cabozantinib
E: Everolimus
H: HAIC (hepatic arterial infusion chemotherapy)
Le: Lenvatinib
AEG: AEG35156
LDF: a Chinese herbal formula
T: cTACE (conventional transarterial chemoembolization)
Si: Sintilimab
Bb: IBI305 (a Bevacizumab biosimilar)
Te: tepotinib
Re: resminostat
Er: erlotinib
Cr: cryoRx (cryotherapy)
N: Nivolumab
R: Radiotherapy
