# Supplementary material for: Comparison of efficacy and safety of adjuvant therapies versus sorafenib in hepatocellular carcinoma: a systematic review and network meta-analysis
Source: Front Pharmacol. 2025 Mar 3;16:1502931. doi: 10.3389/fphar.2025.1502931 (PMC11911332; doi:10.3389/fphar.2025.1502931)
Supplement: Supplementary file 1 [file Table1.docx]

**Supplementary Table 1.** The search strategies used in the databases

| **Database** | **Search strategy** |
| --- | --- |
| PubMed | ((("Carcinoma, Hepatocellular"[Mesh]) OR (((((((((((((((((((Carcinoma, Hepatocellular[Title/Abstract]) OR (Carcinomas, Hepatocellular[Title/Abstract])) OR (Hepatocellular Carcinomas[Title/Abstract])) OR (Liver Cell Carcinoma, Adult[Title/Abstract])) OR (Liver Cancer, Adult[Title/Abstract])) OR (Adult Liver Cancer[Title/Abstract])) OR (Adult Liver Cancers[Title/Abstract])) OR (Cancer, Adult Liver[Title/Abstract])) OR (Cancers, Adult Liver[Title/Abstract])) OR (Liver Cancers, Adult[Title/Abstract])) OR (Liver Cell Carcinoma[Title/Abstract])) OR (Carcinoma, Liver Cell[Title/Abstract])) OR (Carcinomas, Liver Cell[Title/Abstract])) OR (Cell Carcinoma, Liver[Title/Abstract])) OR (Cell Carcinomas, Liver[Title/Abstract])) OR (Liver Cell Carcinomas[Title/Abstract])) OR (Hepatocellular Carcinoma[Title/Abstract])) OR (Hepatoma[Title/Abstract])) OR (Hepatomas[Title/Abstract]))) AND (("Sorafenib"[Mesh]) OR (((((((((((((((Sorafenib[Title/Abstract]) OR (Nexavar[Title/Abstract])) OR (BAY 43-9006[Title/Abstract])) OR (BAY 43 9006[Title/Abstract])) OR (BAY 439006[Title/Abstract])) OR (Sorafenib N-Oxide[Title/Abstract])) OR (Sorafenib N Oxide[Title/Abstract])) OR (BAY-673472[Title/Abstract])) OR (BAY 673472[Title/Abstract])) OR (BAY 545-9085[Title/Abstract])) OR (BAY 545 9085[Title/Abstract])) OR (BAY 5459085[Title/Abstract])) OR (BAY-545-9085[Title/Abstract])) OR (BAY5459085[Title/Abstract])) OR (Sorafenib Tosylate[Title/Abstract])))) AND (randomized controlled trial[Publication Type] OR randomized[Title/Abstract] OR placebo[Title/Abstract]) |
| EMBASE | 1. 'liver cell carcinoma'/exp 2. 'carcinoma, hepatocellular':ab,ti 3. 'hepatocellular carcinomas':ab,ti 4. 'liver cell carcinoma, adult':ab,ti 5. 'liver cancer, adult':ab,ti 6. 'adult liver cancer':ab,ti 7. 'adult liver cancers':ab,ti 8. 'cancer, adult liver':ab,ti 9. 'cancers, adult liver':ab,ti 10. 'carcinomas, hepatocellular':ab,ti 11. 'liver cancers, adult':ab,ti 12. 'liver cell carcinoma':ab,ti 13. 'carcinoma, liver cell':ab,ti 14. 'carcinomas, liver cell':ab,ti 15. 'cell carcinoma, liver':ab,ti 16. 'cell carcinomas, liver':ab,ti 17. 'liver cell carcinomas':ab,ti 18. 'hepatocellular carcinoma':ab,ti 19. 'hepatoma':ab,ti 20. 'hepatomas':ab,ti 21. #1 OR #2 OR #3 OR #4 OR #5 OR #6 OR #7 OR #8 OR #9 OR #10 OR #11 OR #12 OR #13 OR #14 OR #15 OR #16 OR #17 OR #18 OR #19 OR #20 22. 'sorafenib'/exp 23. 'sorafenib':ab,ti 24. 'nexavar':ab,ti 25. 'bay 43-9006':ab,ti 26. 'bay 43 9006':ab,ti 27. 'bay 439006':ab,ti 28. 'sorafenib n-oxide':ab,ti 29. 'sorafenib n oxide':ab,ti 30. 'bay-673472':ab,ti 31. 'bay 673472':ab,ti 32. 'bay 545-9085':ab,ti 33. 'bay 545 9085':ab,ti 34. 'bay 5459085':ab,ti 35. 'bay-545-9085':ab,ti 36. 'bay5459085':ab,ti 37. 'sorafenib tosylate':ab,ti 38. #22 OR #23 OR #24 OR #25 OR #26 OR #27 OR #28 OR #29 OR #30 OR #31 OR #32 OR #33 OR #34 OR #35 OR #36 OR #37 39. 'random':ab,ti OR 'placebo':ab,ti OR 'double-blind':ab,ti 40. #21 AND #38 AND #39 |
| Web of Science | 1. (((((((((((((((((((TS=(Carcinoma, Hepatocellular)) OR TS=(Carcinomas, Hepatocellular)) OR TS=(Hepatocellular Carcinomas)) OR TS=(Liver Cell Carcinoma, Adult)) OR TS=(Liver Cancer, Adult)) OR TS=(Adult Liver Cancer)) OR TS=(Adult Liver Cancers)) OR TS=(Cancer, Adult Liver)) OR TS=(Cancers, Adult Liver)) OR TS=(Liver Cancers, Adult)) OR TS=(Carcinoma, Liver Cell)) OR TS=(Liver Cell Carcinoma)) OR TS=(Carcinomas, Liver Cell)) OR TS=(Cell Carcinoma, Liver)) OR TS=(Cell Carcinomas, Liver)) OR TS=(Liver Cell Carcinomas)) OR TS=(Hepatocellular Carcinoma)) OR TS=(Hepatoma)) OR TS=(Hepatomas)) 2. (((((((((((((((TS=(Sorafenib)) OR TS=(Nexavar)) OR TS=(BAY 43-9006)) OR TS=(BAY 43 9006)) OR TS=(BAY 439006)) OR TS=(Sorafenib N-Oxide)) OR TS=(Sorafenib N Oxide)) OR TS=(BAY-673472)) OR TS=(BAY 673472)) OR TS=(BAY 545-9085)) OR TS=(BAY 545 9085)) OR TS=(BAY 5459085)) OR TS=(BAY-545-9085)) OR TS=(BAY5459085)) OR TS=(Sorafenib Tosylate)) 3. (((((TS=(random)) OR TS=(placebo)) OR TS=(double-blind)) OR TS=(randomized controlled trial)) OR TS=(randomized)) 4. (#3 AND #2 AND #1) |
| Cochrane Library | 1. MeSH descriptor: [Carcinoma, Hepatocellular] explode all trees 2. (Carcinoma, Hepatocellular):ti,ab,kw OR (Carcinomas, Hepatocellular):ti,ab,kw OR (Hepatocellular Carcinomas):ti,ab,kw OR (Liver Cell Carcinoma, Adult):ti,ab,kw OR (Liver Cancer, Adult):ti,ab,kw 3. (Adult Liver Cancer):ti,ab,kw OR (Adult Liver Cancers):ti,ab,kw OR (Cancer, Adult Liver):ti,ab,kw OR (Cancers, Adult Liver):ti,ab,kw OR (Liver Cancers, Adult):ti,ab,kw 4. (Liver Cell Carcinoma):ti,ab,kw OR (Carcinoma, Liver Cell):ti,ab,kw OR (Carcinomas, Liver Cell):ti,ab,kw OR (Cell Carcinoma, Liver):ti,ab,kw OR (Cell Carcinomas, Liver):ti,ab,kw 5. (Liver Cell Carcinomas):ti,ab,kw OR (Hepatocellular Carcinoma):ti,ab,kw OR (Hepatoma):ti,ab,kw OR (Hepatomas):ti,ab,kw 6. #1 OR #2 OR #3 OR #4 OR #5 7. MeSH descriptor: [Sorafenib] explode all trees 8. (Sorafenib):ti,ab,kw OR (Nexavar):ti,ab,kw OR (BAY 43 9006):ti,ab,kw OR (BAY 439006):ti,ab,kw OR (Sorafenib N-Oxide):ti,ab,kw 9. (Sorafenib N Oxide):ti,ab,kw OR (BAY-673472):ti,ab,kw OR (BAY 673472):ti,ab,kw OR (BAY 545 9085):ti,ab,kw 10. #7 OR #8 OR #9 11. #6 AND #10 |

**Supple****mentary Table 2.** Characteristics of the included studies

| **Study** | **Country** | **Sample size** | **Male** | **Age** | **Intervention** | **Outcome measure** |
| --- | --- | --- | --- | --- | --- | --- |
| Abdel^[1]^  2013 | USA | S: 26  Cap: 26 | NR | S: 53.5  Cap: 59.5 | Sorafenib:400mg twice daily  Capecitabine: 1000mg/m^2^ twice daily | PFS OS ORR DCR AEs |
| Abou-Alfa^[2]^  2019 | USA | S:176  S+D: 180 | S: 153  S+D: 153 | S: 61.5  S+D: 62.0 | Sorafenib:400mg twice daily  Sorafenib+ Doxorubicin: 400mg+60-360 mg/m^2^ twice daily | PFS OS ORR DCR AEs |
| Assenat^[3]^  2019 | France | S:44  S+G:39 | S:38  S+G:36 | S:62  S+G:65 | Sorafenib:400mg twice daily  Sorafenib+GEMOX: 400mg twice daily+G1000mg/m^2^ 100min (day1) & O100mg/m^2^ 2h (day2) (day 1=day14/2 weeks) | AEs ORR OS PFS DCR |
| Azim^[4]^  2018 | Egypt | S:38  S+U:36 | S:34  S+U:31 | S:58.5  S+U:59.0 | Sorafenib: 400mg twice daily continuously  Sorafenib + UFT: 400mg twice daily continuously+U125 mg/m^2^ twice daily for 28days (repeated on day 36/5 weeks) | DCR ORR AEs OS PFS |
| Blanc.J.F^[5]^  2021 | France | S:41  P:39  S+P:40 | S:37  P:34  S+P:35 | S:67  P:63  S+P:66 | Sorafenib: 400mg twice daily continuously (dose reductions according to toxicities)  Pravastain:40mg per day continuously  Sorafenib + Pravastain: 400mg twice daily +40mg per day continuously | OS PFS AEs |
| Cainap^[6]^  2017 | Romania | S:521  L:514 | S:436  L:444 | S:60  L:59 | Sorafenib: 400mg twice daily  Linifanib:17.5mg once daily | OS PFS ORR AEs |
| Cheng^[7]^  2013 | China  (Taiwan) | S:544  Su:530 | S:459  Su:436 | S:59.0  Su:59.0 | Sorafenib: 400mg twice per day (dose reductions following the package insert)  Sunitinib:37.5mg once daily in the morning (12.5mg incremental dose reductions for managing toxicities) | PFS OS AEs |
| Cheng^[8]^  2016 | China  (Taiwan) | S:83  Dov:82 | S:67  Dov:73 | S:56  Dov:56 | Sorafenib: 400mg continuously twice daily  Dovitinib: 500mg/day on a 5 days on, 2days off schedule | AEs OS ORR DCR |
| Cheng^[9]^  2015 | China  (Taiwan) | S: 55  S+Ti2:53 S+Ti6:54 | S: 44  S+Ti2:45 S+Ti6::45 | S:66  S+Ti2:63 S+Ti6::62.5 | Sorafenib: 400mg twice daily  Tigatuzumab2+ Sorafenib: 6mg/kg loading, 2mg/kg/week maintenance +400mg twice daily Tigatuzumab6+ Sorafenib: 6mg/kg loading, 6mg/kg/week maintenance +400mg twice daily | OS ORR AEs |
| Chow.P^[10]^  2018 | Singapore | S:178  Y:182 | S:151  Y:147 | S:57.7  Y:59.5 | Sorafenib: 400mg twice daily  RE: ^90^Y loaded resin microspheres within 35 days of random assignment | DCR PFS AEs OS |
| Ciuleanu^[11]^  2016 | Romania | S:51  S+Ma:50 | S:39  S+Ma:26 | S:64.0  S+Ma:60.0 | Sorafenib: 400mg twice daily per 21-day cycle  Mapatumumab+ Sorafenib:30mg/kg on Day 1 per 21-day cycle + 400mg twice daily per 21-day cycle | AEs PFS OS |
| Finn^[12]^  2020 | China  (Taiwan) | S:165  A+B:336 | S:137  A+B:277 | S:66  A+B:64 | Sorafenib: 400mg twice daily  Atezolizumab+ Bevacizumab: 1200mg+15mg per kilogram of body weight (every 3 weeks) | OS PFS DCR ORR AEs |

Continued Supplementary Table 2. Characteristics of the included studies

| Giorgio^[13]^  2016 | Italy | S:50  S+RFA:49 | S:36  S+RFA:37 | S:72  S+RFA:71 | Sorafenib: 800mg/die. Per O.S  Sorafenib+RFA: 800mg/die. Per O.S+RAF | OS AEs |
| --- | --- | --- | --- | --- | --- | --- |
| Haruna^[14]^  2021 | Japan | S: 22  S+K:22 | S:18  S+K:17 | S:71.9  S+K:72 | Sorafenib: 400mg twice daily  Sorafenib+ vitamin K: 400mg twice daily+15mg three times daily | PFS OS AEs  ORR DCR |
| He^[15]^  2019 | China | S: 122  S+OFL:125 | NR | NR | Sorafenib: 400mg twice daily  Sorafenib+OFL: 400mg twice daily+(85mg/m^2^ O+400mg/m^2^ L+400mg/m^2^ F bolus on day 1, F infusion 2400 mg/m^2^ for 46hours, every 3 weeks) | DCR ORR AEs OS PFS |
| Ikeda^[16]^  2016 | Kashiwa | S: 41  S+SorCDDP: 65 | S: 32  S+SorCDDP: 56 | S: 64  S+SorCDDP: 66 | Sorafenib: 400mg bid  Sorafenib +SorCDDP: 400mg bid+65mg/m^2^ day 1 every 4-6 weeks | OS AEs |
| Johnson^[17]^  2013 | UK | S: 578  Br:577 | S: 484  Br: 483 | S: 60  Br: 61 | Sorafenib: 400mg twice daily  Brivanib: 800mg once daily | OS ORR DCR AEs |
| Jouve^[18]^  2019 | France | S: 161  S+P: 162 | S: 142  S+P: 156 | S: 68  S+P: 68 | Sorafenib: 400mg twice daily  Sorafenib+ Pravastatin: 400mg twice daily+40mg per day (drug intake during the dinner) | OS PFS AEs |
| Kelley^[19]^  2022 | USA | S:217  C:188  C+A:432 | S:186  C:158  C+A:360 | S:64  C:64  C+A:64 | Sorafenib:400mg twice daily  Cabozantinib:60mg once daily  Cabozantinib+Atezolizumab:40mg once daily+ a dose of 1200mg every 3 weeks | PFS OS ORR AEs DCR |
| Koeberle^[20]^  2016 | Switzerland | S:46  S+E:59 | S:40  S+E:48 | S:65  S+E:66 | Sorafenib: 400mg twice daily  Sorafenib+ Everolimus: 400mg twice daily+5mg daily | AEs PFS OS |
| Kondo^[21]^  2019 | Japan | S:33  S+H:35 | S:27  S+H:28 | S:70.9  S+H:72.0 | Sorafenib: 400mg twice daily  Sorafenib+ HAIC:400mg twice daily + a dose of 65mg/m^2^ cisplatin over a period of about 30mins (repeated at an interval of 4 to 6 weeks) | OS ORR DCR AEs |
| Kudo^[22]^  2018 | Japan | S:476  Le:478 | S:401  Le:405 | S:61.2  Le:61.3 | Sorafenib: 400mg twice daily  Lenvatinib: a dose of 12mg per day (for body weight >=60kg) or 8mg per day (for body weight <60kg) | OS PFS DCR ORR AEs |
| Kudo^[23]^  2018 | Japan | S:103  S+H:102 | S:88  S+H:89 | S:68  S+H:69 | Sorafenib: 400mg twice daily on days 1-28  Sorafenib +HAIC: 400mg twice daily on days 1-28+ a dose of 20mg/m^2^ cisplatin per day on days 1 and 8+a dose of 330mg/m^2^ fluorouracil per day on days 1-5 and 8-12 of every 28-day cycle followed by 2 weeks off treatment | DCR ORR OS PFS AEs |
| Lee.F.A.S^[24]^  2016 | China  (Hong Kong) | S:17  S+AEG:31 | S:15  S+AEG:27 | S:54  S+AEG:61 | Sorafenib: 400mg twice daily (3 weeks/each cycle)  Sorafenib+AEG35156: 400mg twice daily+300mg weekly intravenous infusion (3 weeks/each cycle) | OS PFS ORR DCR AEs |
| Liang^[25]^  2020 | China | S:40  LDF:40 | S:26  LDF:29 | S:51  LDF:50 | Sorafenib:0.4g bid  LDF: tid, 100ml/time | OS DCR ORR AEs |

Continued Supplementary Table 2. Characteristics of the included studies

| Park.J.W^[26]^  2019 | Korea | S:169  S+T:170 | S:147  S+T:136 | S:61.3  S+T:60.2 | Sorafenib:600/day (200-400mg twice daily) within 3 days and then 400mg twice daily  Sorafenib +cTACE: 600/day (200-400mg twice daily) within 3 days and then receive first cTACE between 7 and 21days and then resume S after the first cTACE when they met the S treatment initiation criteria | DCR ORR AEs OS PFS |
| --- | --- | --- | --- | --- | --- | --- |
| Ren^[27]^  2021 | China | S:191  Si+Bb:380 | S:171  Si+Bb:334 | S:54  Si+Bb:53 | Sorafenib: 400mg twice daily  Sintilimab+IBI305: 200 mg of Si intravenously over 60 min, 15 mg/kg of Bb intravenously over 90 min, 3 weeks | ORR OS DCR PFS AEs |
| Ricke^[28]^  2015 | Germany | S:20  S+Y:20 | S:14  S+Y:16 | S:68.5  S+Y:71.5 | Sorafenib:200 mg twice daily for 1 week before increasing the dose to sorafenib 400 mg twice daily  Sorafenib+^90^Y-radioembolization: a sequential lobar approach and on day 3 after the last radioembolization procedure, recieve same S | AEs |
| Ricke^[29]^  2019 | Germany | S:208  S+Y:216 | S:177  S+Y:181 | S:66  S+Y:66 | Sorafenib: 200 mg twice daily for 1 week before increasing the dose to sorafenib 400 mg twice daily  Sorafenib+SY: 200 mg twice daily for 1 week before increasing the dose to sorafenib 400 mg twice daily+ SIRT with ^90^Y resin microspheres | AEs OS |
| Ryoo^[30]^  2021 | Korea | S:37  Te:38 | S:34  Te:37 | S:54  Te:59 | sorafenib: 400 mg twice daily  tepotinib: tepotinib hydrochloride hydrate 300mg, 500mg, 1000mg 21-day cycles | PFS OS AEs ORR DCR |
| Shorbagy^[31]^  2021 | Egypt | S:40  S+M:40 | S:31  S+M:30 | NR | Sorafenib: 400mg twice daily  Sorafenib+ Metformin: 400mg+500mg twice daily | OS AEs |
| Tak^[32]^  2018 | Korea | S:84  S+Re:83 | S:73  S+Re:66 | S:62  S+Re:65 | sorafenib: 400 mg twice daily  sorafenib+resminostat: 400 mg twice daily+400mg once daily on days 1 to 5 of a 14-day treatment cycle | OS ORR DCR AEs |
| Thomas^[33]^  2018 | USA | S:43  B+Er:47 | NR | S:61  B+Er:61 | sorafenib: 400 mg twice daily  Bevacizumab+erlotinib:10mg/kg B every 14 days+150mg E daily | OS AEs |
| Vilgrain^[34]^  2017 | France | S:222  Y:237 | S:202  Y:212 | S:65  Y:66 | sorafenib: 400 mg twice daily  SY: SIRT with ^90^Y resin microspheres 2-5 weeks after randomisation | PFS OS ORR DCR AEs |
| Yang^[35]^  2012 | China | S:52  S+Cr:52 | S:47  S+Cr:48 | S:52.6  S+Cr:51.2 | sorafenib: 400 mg twice daily for at least 8 weeks  sorafenib+cryoRx: 400 mg twice daily for at least 8 weeks+argon-helium gas-based CRYOcare system&cryoprobes | OS DCR ORR AEs |
| Yau^[36]^  2022 | China  (Hong Kong) | S:372  N:371 | S:317  N:314 | NR | sorafenib: 400 mg twice daily  Nivolumab:240mg intravenously every 2 weeks | OS PFS ORR DCR AEs |
| Yen^[37]^  2018 | China  (Taiwan) | S:32  N:63 | S:26  N:57 | S:62.0  N:58.0 | sorafenib: 400 mg twice daily continuously in 28-day cycles  Nivolumab: 200 mg twice daily | PFS OS AEs |

Continued Supplementary Table 2. Characteristics of the included studies

| Yoon^[38]^  2018 | Korea | S:45  T+R:45 | S:39  T+R:38 | S:55  T+R:55 | sorafenib: 400 mg twice daily  Transarterial chemoembolization +Radiotherapy: T every 6 weeks+ R within 3 weeks after first T, maximum 45Gy with the fraction size of 2.5 to 3 Gy | DCR ORR PFS OS AEs |
| --- | --- | --- | --- | --- | --- | --- |
| Zheng^[39]^  2022 | China | S: 32  S+H: 32 | S: 31  S+H: 30 | S: 55  S+H: 56 | sorafenib: 400 mg twice daily  sorafenib+HAIC: 400 mg twice daily+3cir-OFF HAIC 35mg/m^2^ oxaliplatin hour 0-2 followed by 600mg/m^2^ 5-fluorouracil hour 2-24, day 1-3 | OS PFS ORR DCR AEs |
| Zhu^[40]^  2015 | France | S:358  S+Er: 362 | S:286  S+Er:295 | S:60.0  S+Er:60.5 | sorafenib: 400 mg twice one day+150mg once a day  sorafenib+Erlotinib: 400 mg twice one day+150mg once a day | OS ORR DCR AEs |

**Supplementary Table 3.** OS league table

**Supplementary Table 4.** PFS league table

**Supplementary Table 5.** ORR league table

**Supplementary Table 6.** DCR league table

**Supplementary** **Table 7.** AEs league table

**Supplementary Table 8.** SAE league table

**Reference**

[1] O. Abdel-Rahman, M. Abdel-Wahab, M. Shaker, S. Abdel-Wahab, M. Elbassiony, and M. Ellithy, “Sorafenib versus capecitabine in the management of advanced hepatocellular carcinoma”, *Med. Oncol.*, vol. 30, no. 3, p. 655, Sep. 2013, doi: 10.1007/s12032-013-0655-z.

[2] G. K. Abou-Alfa *et al.*, “Assessment of treatment with sorafenib plus doxorubicin vs sorafenib alone in patients with advanced hepatocellular carcinoma: phase 3 CALGB 80802 randomized clinical trial”, *JAMA Oncol.*, vol. 5, no. 11, p. 1582, Nov. 2019, doi: 10.1001/jamaoncol.2019.2792.

[3] 《Assenat et al._2019_Sorafenib alone vs. sorafenib plus GEMOX as 1st-line treatment for advanced HCC the phase II random.pdf》.

[4] 《Azim-2018-Sorafenib plus tegafur–uracil (UFT).pdf》.

[5] J.-F. Blanc *et al.*, “Phase 2 trial comparing sorafenib, pravastatin, their combination or supportive care in HCC with Child–Pugh B cirrhosis”, *Hepatol. Int.*, vol. 15, no. 1, pp. 93–104, Feb. 2021, doi: 10.1007/s12072-020-10120-3.

[6] C. Cainap *et al.*, “Linifanib versus sorafenib in patients with advanced hepatocellular carcinoma: results of a randomized phase III trial”, *J. Clin. Oncol.*, vol. 33, no. 2, pp. 172–179, Jan. 2015, doi: 10.1200/JCO.2013.54.3298.

[7] A.-L. Cheng *et al.*, “Sunitinib versus sorafenib in advanced hepatocellular cancer: results of a randomized phase III trial”, *J. Clin. Oncol.*, vol. 31, no. 32, pp. 4067–4075, Nov. 2013, doi: 10.1200/JCO.2012.45.8372.

[8] A. Cheng *et al.*, “Randomized, open‐label phase 2 study comparing frontline dovitinib versus sorafenib in patients with advanced hepatocellular carcinoma”, *Hepatology*, vol. 64, no. 3, pp. 774–784, Sep. 2016, doi: 10.1002/hep.28600.

[9] A.-L. Cheng *et al.*, “Safety and efficacy of tigatuzumab plus sorafenib as first-line therapy in subjects with advanced hepatocellular carcinoma: a phase 2 randomized study”, *J. Hepatol.*, vol. 63, no. 4, pp. 896–904, Oct. 2015, doi: 10.1016/j.jhep.2015.06.001.

[10] P. K. H. Chow *et al.*, “SIRveNIB: selective internal radiation therapy versus sorafenib in Asia-pacific patients with hepatocellular carcinoma”, *J. Clin. Oncol.*, vol. 36, no. 19, pp. 1913–1921, Jul. 2018, doi: 10.1200/JCO.2017.76.0892.

[11] T. Ciuleanu *et al.*, “A randomized, double-blind, placebo-controlled phase II study to assess the efficacy and safety of mapatumumab with sorafenib in patients with advanced hepatocellular carcinoma”, *Ann. Oncol.*, vol. 27, no. 4, pp. 680–687, Apr. 2016, doi: 10.1093/annonc/mdw004.

[12] R. S. Finn *et al.*, “Atezolizumab plus bevacizumab in unresectable hepatocellular carcinoma”, *N. Engl. J. Med.*, vol. 382, no. 20, pp. 1894–1905, May 2020, doi: 10.1056/NEJMoa1915745.

[13] A. Giorgio *et al.*, “Sorafenib Combined with Radio-frequency Ablation Compared with Sorafenib Alone in Treatment of Hepatocellular Carcinoma Invading Portal Vein: A Western Randomized Controlled Trial”, *Anticancer Res.*, vol. 36, no. 11, pp. 6179–6184, Nov. 2016, doi: 10.21873/anticanres.11211.

[14] Y. Haruna, T. Yakushijin, and S. Kawamoto, “Efficacy and safety of sorafenib plus vitamin K treatment for hepatocellular carcinoma: a phase II, randomized study”, *Cancer Med.*, vol. 10, no. 3, pp. 914–922, Feb. 2021, doi: 10.1002/cam4.3674.

[15] M. He *et al.*, “Sorafenib plus hepatic arterial infusion of oxaliplatin, fluorouracil, and leucovorin vs sorafenib alone for hepatocellular carcinoma with portal vein invasion: a randomized clinical trial”, *JAMA Oncol.*, vol. 5, no. 7, p. 953, Jul. 2019, doi: 10.1001/jamaoncol.2019.0250.

[16] M. Ikeda *et al.*, “Sorafenib plus hepatic arterial infusion chemotherapy with cisplatin versus sorafenib for advanced hepatocellular carcinoma: randomized phase II trial”, *Ann. Oncol.*, vol. 27, no. 11, pp. 2090–2096, Nov. 2016, doi: 10.1093/annonc/mdw323.

[17] P. J. Johnson *et al.*, “Brivanib versus sorafenib As first-line therapy in patients with unresectable, advanced hepatocellular carcinoma: results from the randomized phase III BRISK-FL study”, *J. Clin. Oncol.*, vol. 31, no. 28, pp. 3517–3524, Oct. 2013, doi: 10.1200/JCO.2012.48.4410.

[18] J.-L. Jouve *et al.*, “Pravastatin combination with sorafenib does not improve survival in advanced hepatocellular carcinoma”, *J. Hepatol.*, vol. 71, no. 3, pp. 516–522, Sep. 2019, doi: 10.1016/j.jhep.2019.04.021.

[19] R. K. Kelley *et al.*, “Cabozantinib plus atezolizumab versus sorafenib for advanced hepatocellular carcinoma (COSMIC-312): a multicentre, open-label, randomised, phase 3 trial”, *Lancet Oncol.*, vol. 23, no. 8, pp. 995–1008, Aug. 2022, doi: 10.1016/S1470-2045(22)00326-6.

[20] D. Koeberle *et al.*, “Sorafenib with or without everolimus in patients with advanced hepatocellular carcinoma (HCC): a randomized multicenter, multinational phase II trial (SAKK 77/08 and SASL 29)”, *Ann. Oncol.*, vol. 27, no. 5, pp. 856–861, May 2016, doi: 10.1093/annonc/mdw054.

[21] M. Kondo *et al.*, “Randomized, phase II trial of sequential hepatic arterial infusion chemotherapy and sorafenib versus sorafenib alone as initial therapy for advanced hepatocellular carcinoma: SCOOP-2 trial”, *BMC Cancer*, vol. 19, no. 1, p. 954, Dec. 2019, doi: 10.1186/s12885-019-6198-8.

[22] 《Kudo-2018-Lenvatinib versus sorafenib in first.pdf》.

[23] M. Kudo *et al.*, “Sorafenib plus low-dose cisplatin and fluorouracil hepatic arterial infusion chemotherapy versus sorafenib alone in patients with advanced hepatocellular carcinoma (SILIUS): a randomised, open label, phase 3 trial”, *Lancet Gastroenterol. Hepatol.*, vol. 3, no. 6, pp. 424–432, Jun. 2018, doi: 10.1016/S2468-1253(18)30078-5.

[24] F. A. S. Lee *et al.*, “Randomized Phase II Study of the X-linked Inhibitor of Apoptosis (XIAP) Antisense AEG35156 in Combination With Sorafenib in Patients With Advanced Hepatocellular Carcinoma (HCC)”, *Am. J. Clin. Oncol.*, vol. 39, no. 6, pp. 609–613, Dec. 2016, doi: 10.1097/COC.0000000000000099.

[25] X. Liang and X.-Y. Hu, “The efficacy and safety of LDF, a chinese herbal formula, compared with sorafenib for the treatment of advanced hepatocellular carcinoma in chinese patients: a retrospective cohort study”.

[26] J.-W. Park *et al.*, “Sorafenib with or without concurrent transarterial chemoembolization in patients with advanced hepatocellular carcinoma: the phase III STAH trial”, *J. Hepatol.*, vol. 70, no. 4, pp. 684–691, Apr. 2019, doi: 10.1016/j.jhep.2018.11.029.

[27] Z. Ren *et al.*, “Sintilimab plus a bevacizumab biosimilar (IBI305) versus sorafenib in unresectable hepatocellular carcinoma (ORIENT-32): a randomised, open-label, phase 2–3 study”, *Lancet Oncol.*, vol. 22, no. 7, pp. 977–990, Jul. 2021, doi: 10.1016/S1470-2045(21)00252-7.

[28] J. Ricke *et al.*, “Safety and toxicity of radioembolization plus Sorafenib in advanced hepatocellular carcinoma: analysis of the European multicentre trial SORAMIC”, *Liver Int.*, vol. 35, no. 2, pp. 620–626, Feb. 2015, doi: 10.1111/liv.12622.

[29] J. Ricke *et al.*, “Impact of combined selective internal radiation therapy and sorafenib on survival in advanced hepatocellular carcinoma”, *J. Hepatol.*, vol. 71, no. 6, pp. 1164–1174, Dec. 2019, doi: 10.1016/j.jhep.2019.08.006.

[30] B.-Y. Ryoo *et al.*, “Randomised phase 1b/2 trial of tepotinib vs sorafenib in asian patients with advanced hepatocellular carcinoma with MET overexpression”, *Br. J. Cancer*, vol. 125, no. 2, pp. 200–208, Jul. 2021, doi: 10.1038/s41416-021-01380-3.

[31] S. El Shorbagy *et al.*, “Prognostic significance of VEGF and HIF-1 α in hepatocellular carcinoma patients receiving sorafenib versus metformin sorafenib combination”, *J. Gastrointest. Cancer*, vol. 52, no. 1, pp. 269–279, Mar. 2021, doi: 10.1007/s12029-020-00389-w.

[32] W. Y. Tak *et al.*, “Phase I/II study of first-line combination therapy with sorafenib plus resminostat, an oral HDAC inhibitor, versus sorafenib monotherapy for advanced hepatocellular carcinoma in east Asian patients”, *Invest. New Drugs*, vol. 36, no. 6, pp. 1072–1084, Dec. 2018, doi: 10.1007/s10637-018-0658-x.

[33] M. B. Thomas *et al.*, “A randomized phase II open-label multi-institution study of the combination of bevacizumab and erlotinib compared to sorafenib in the first-line treatment of patients with advanced hepatocellular carcinoma”, *Oncology*, vol. 94, no. 6, pp. 329–339, 2018, doi: 10.1159/000485384.

[34] V. Vilgrain *et al.*, “Efficacy and safety of selective internal radiotherapy with yttrium-90 resin microspheres compared with sorafenib in locally advanced and inoperable hepatocellular carcinoma (SARAH): an open-label randomised controlled phase 3 trial”, *Lancet Oncol.*, vol. 18, no. 12, pp. 1624–1636, Dec. 2017, doi: 10.1016/S1470-2045(17)30683-6.

[35] Y. Yang *et al.*, “Cryotherapy is associated with improved clinical outcomes of sorafenib for the treatment of advanced hepatocellular carcinoma”, *Exp. Ther. Med.*, vol. 3, no. 2, pp. 171–180, Feb. 2012, doi: 10.3892/etm.2011.398.

[36] T. Yau *et al.*, “Nivolumab versus sorafenib in advanced hepatocellular carcinoma (CheckMate 459): a randomised, multicentre, open-label, phase 3 trial”, *Lancet Oncol.*, vol. 23, no. 1, pp. 77–90, Jan. 2022, doi: 10.1016/S1470-2045(21)00604-5.

[37] C.-J. Yen *et al.*, “A phase I/randomized phase II study to evaluate the safety, pharmacokinetics, and efficacy of nintedanib versus sorafenib in asian patients with advanced hepatocellular carcinoma”, *Liver Cancer*, vol. 7, no. 2, pp. 165–178, 2018, doi: 10.1159/000486460.

[38] S. M. Yoon *et al.*, “Efficacy and Safety of Transarterial Chemoembolization Plus External Beam Radiotherapy vs Sorafenib in Hepatocellular Carcinoma With Macroscopic Vascular Invasion: A Randomized Clinical Trial”, *JAMA Oncol.*, vol. 4, no. 5, p. 661, May 2018, doi: 10.1001/jamaoncol.2017.5847.

[39] K. Zheng *et al.*, “Sorafenib plus hepatic arterial infusion chemotherapy versus sorafenib for hepatocellular carcinoma with major portal vein tumor thrombosis: a randomized trial”, *Radiology*, vol. 303, no. 2, pp. 455–464, May 2022, doi: 10.1148/radiol.211545.

[40] A. X. Zhu *et al.*, “SEARCH: a phase III, randomized, double-blind, placebo-controlled trial of sorafenib plus erlotinib in patients with advanced hepatocellular carcinoma”, *J. Clin. Oncol.*, vol. 33, no. 6, pp. 559–566, Feb. 2015, doi: 10.1200/JCO.2013.53.7746.
