# Supplementary material for: Comparison of efficacy and safety of adjuvant therapies versus sorafenib in hepatocellular carcinoma: a systematic review and network meta-analysis
Source: Front Pharmacol. 2025 Mar 3;16:1502931. doi: 10.3389/fphar.2025.1502931 (PMC11911332; doi:10.3389/fphar.2025.1502931)
Supplement: Supplementary file 2 [file DataSheet1.docx]

**Supplementary Figure S1.** Risk of bias summary

**
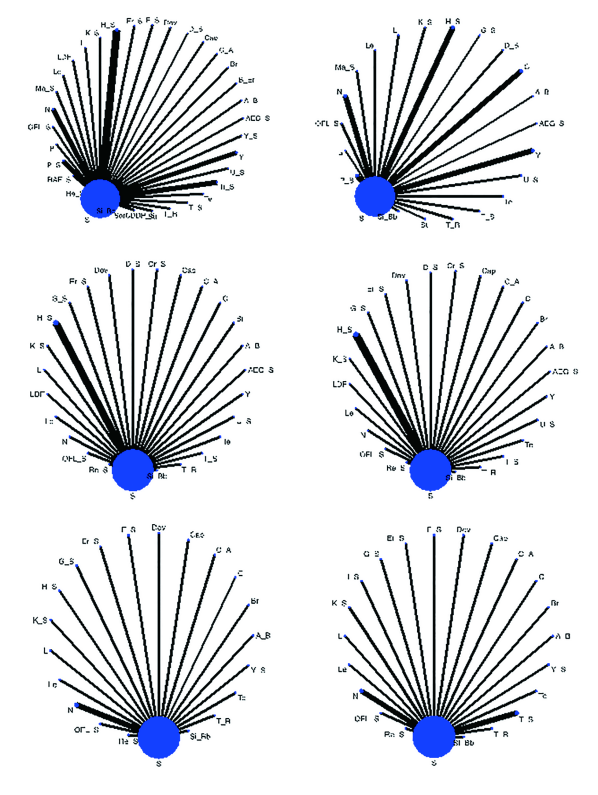
**

**Supplementary Figure S2.** A1 is the network plot of OS; A2 is the network plot of PFS; A3 is the network plot of ORR; A4 is the network plot of DCR; A5 the network plot of AEs; A6 is the network plot of SAE


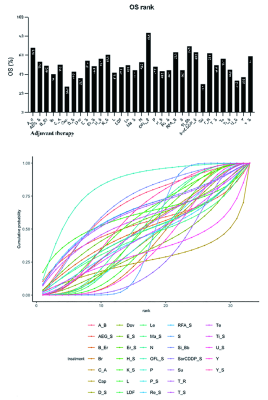


**Supplementary Figure S3.** OS SUCRA rank


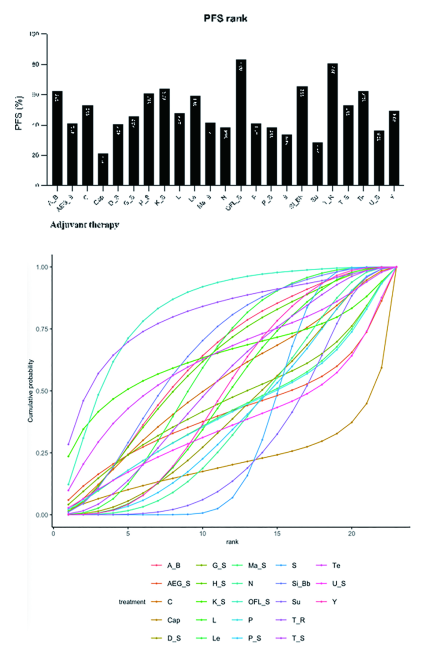


**Supplementary Figure S4.** PFS SUCRA rank


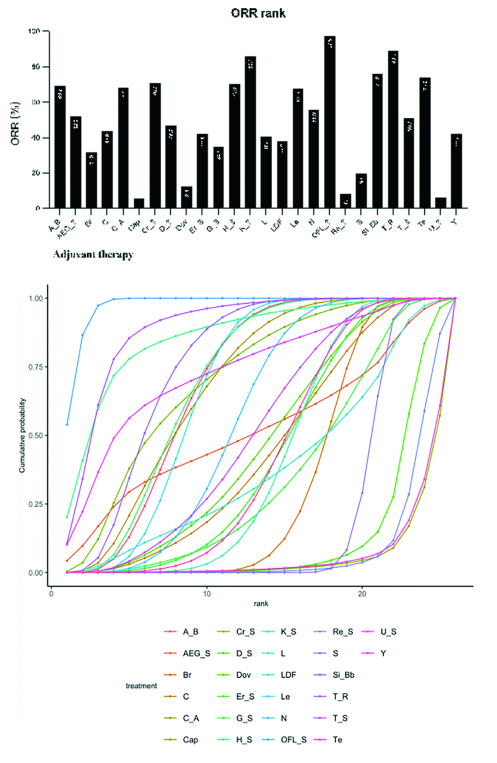


**Supplementary Figure S5.** ORR SUCRA rank


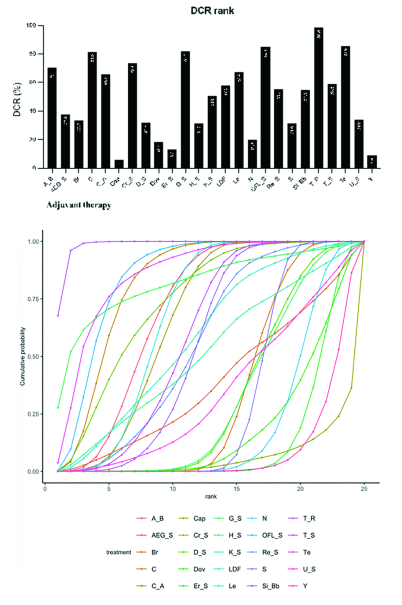


**Supplementary Figure S6.** DCR SUCRA rank


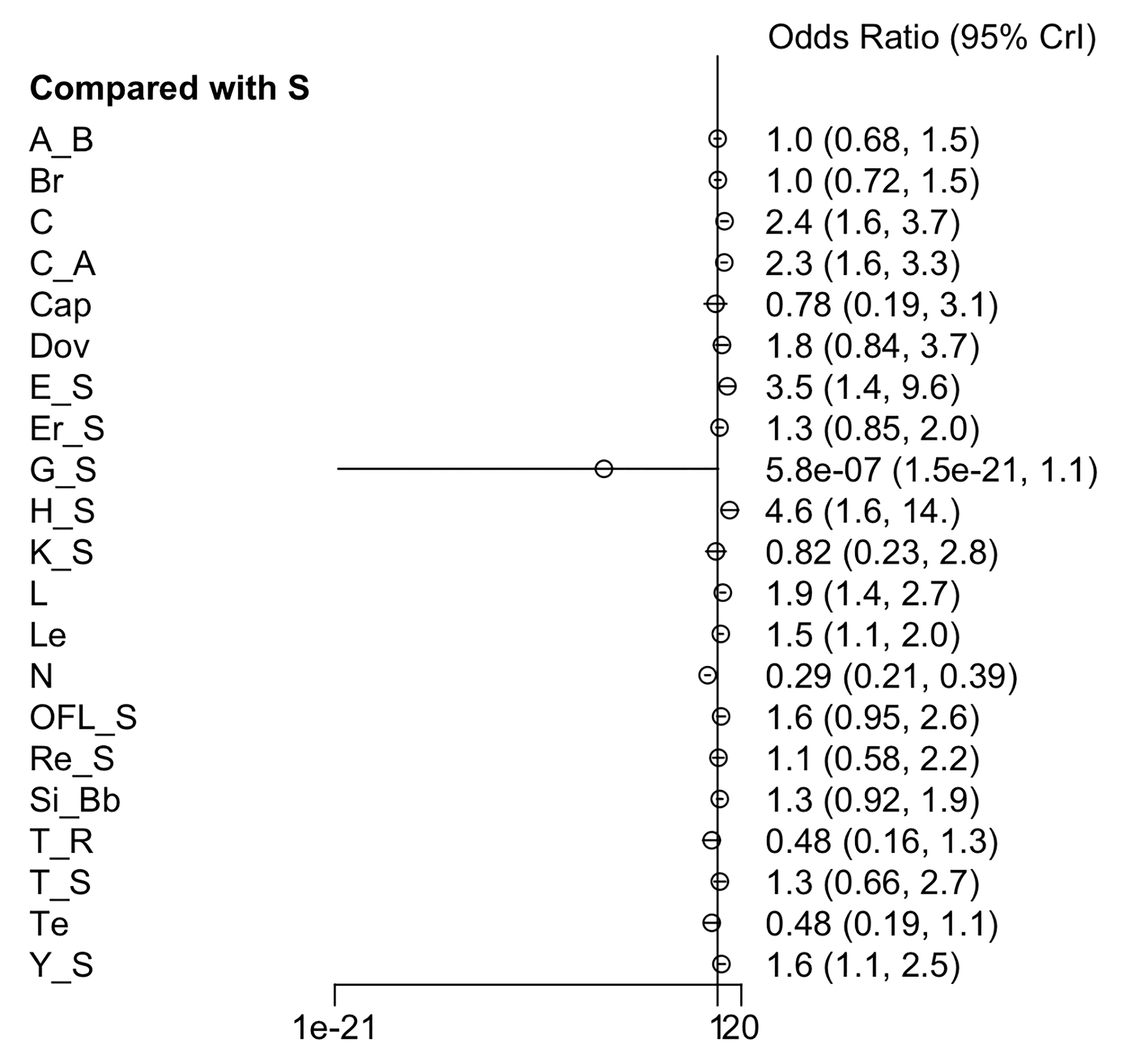


**Supplementary Figure S7.** Forest plot on SAE


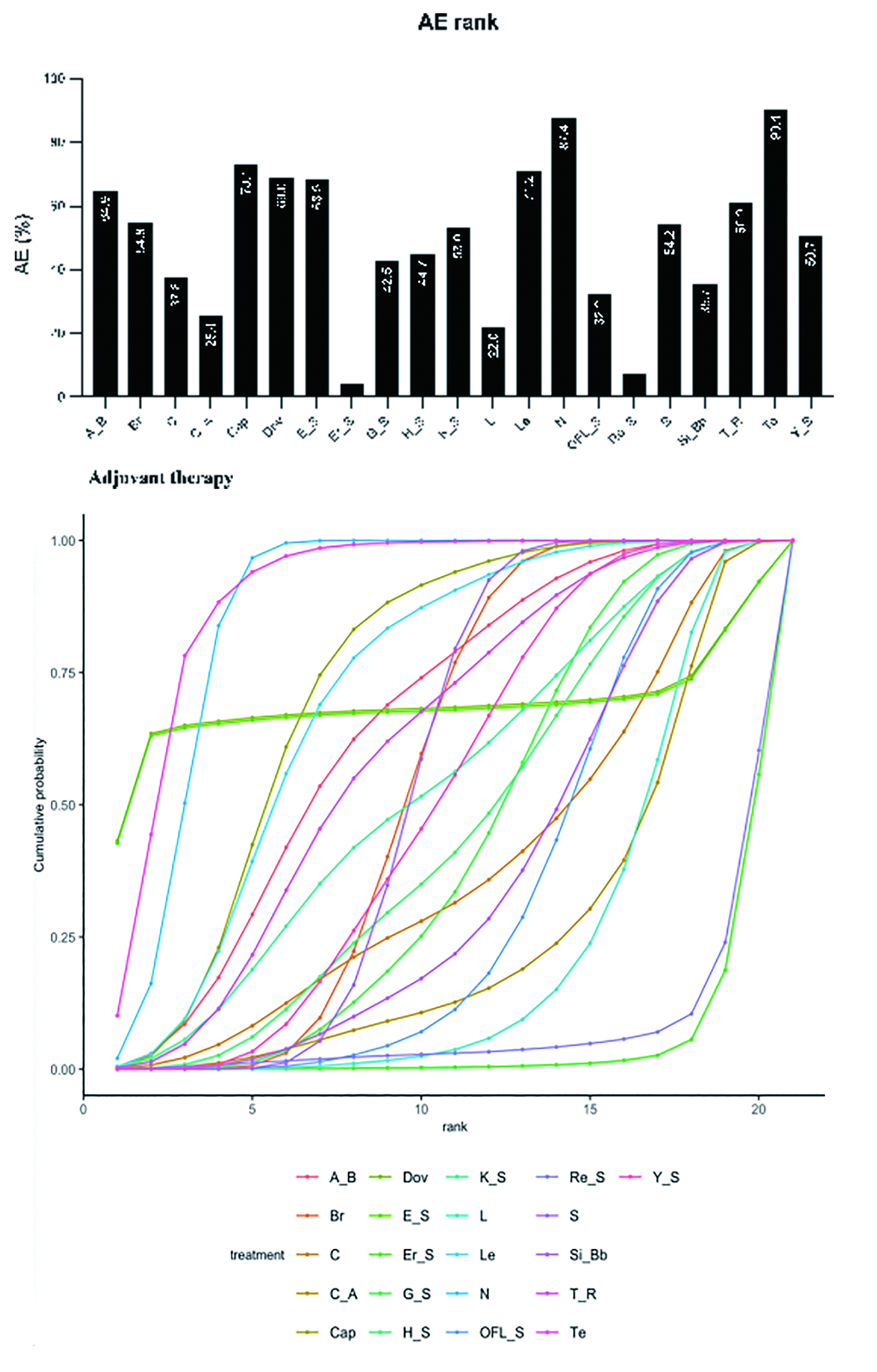


**Supplementary Figure S8**. AEs SUCRA rank


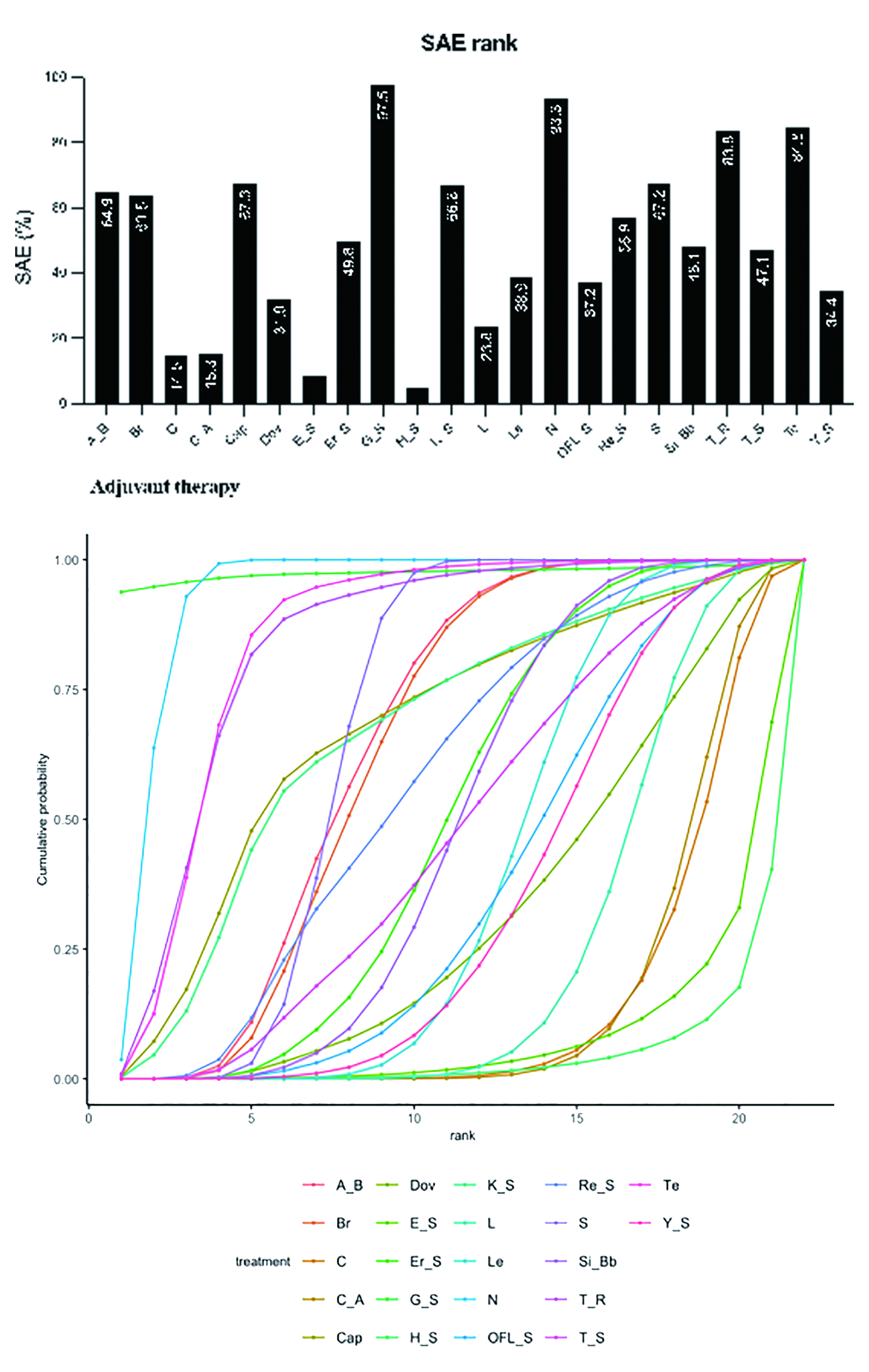


**Supplementary Figure S9**. SAE SUCRA rank

**Supplementary Figure S10.** OS funnel plot

**Supplementary Figure S11.** PFS funnel plot

**Supplementary Figure S12.** ORR funnel plot

**Reference:**

| A: AEG_S | N: L |
| --- | --- |
| B: A_B | O: LDF |
| C: Br | P: Le |
| D: C | Q: N |
| E: C_A | R: OFL_S |
| F: Cap | S: Re_S |
| G: Cr_S | T: S |
| H: D_S | U: Si_Bb |
| I: Dov | V: T_R |
| J: Er_S | W: T_S |
| K: G_S | X: Te |
| L: H_S | Y: U_S |
| M: K_S | Z: Y |

**Supplementary Figure S13.** DCR funnel plot

**Reference:**

| A: AEG_S | N: LDF |
| --- | --- |
| B: A_B | O: Le |
| C: Br | P: N |
| D: C | Q: OFL_S |
| E: C_A | R: Re_S |
| F: Cap | S: S |
| G: Cr_S | T: Si_Bb |
| H: D_S | U: T_R |
| I: Dov | V: T_S |
| J: Er_S | W: Te |
| K: G_S | X: U_S |
| L: H_S | Y: Y |
| M: K_S |  |

**Supplementary Figure S14.** AEs funnel plot

**Reference:**

| A: A_B | L: L |
| --- | --- |
| B: Br | M: Le |
| C: C | N: N |
| D: C_A | O: OFL_S |
| E: Cap | P: Re_S |
| F: Dov | Q: S |
| G: E_S | R: Si_Bb |
| H: Er_S | S: T_R |
| I: G_S | T: Te |
| J: H_S | U: Y_S |
| K: K_S |  |

**Supplementary Figure S15.** SAE funnel plot

**Reference:**

| A: A_B | L: L |
| --- | --- |
| B: Br | M: Le |
| C: C | N: N |
| D: C_A | O: OFL_S |
| E: Cap | P: Re_S |
| F: Dov | Q: S |
| G: E_S | R: Si_Bb |
| H: Er_S | S: T_R |
| I: G_S | T: T_S |
| J: H_S | U: Te |
| K: K_S | V: Y_S |
